# Cost-Effectiveness of Elbasvir/Grazoprevir for the Treatment of Chronic Hepatitis C: A Systematic Review

**DOI:** 10.3389/fpubh.2022.836986

**Published:** 2022-05-13

**Authors:** Jinyu Liu, Min Guo, Lei Ke, Ruxu You

**Affiliations:** ^1^Department of Pharmacy, Tongji Medical College, Tongji Hospital, Huazhong University of Science and Technology, Wuhan, China; ^2^Department of Pharmacy, Tongji Medical College, Union Hospital, Huazhong University of Science and Technology, Wuhan, China

**Keywords:** cost-effectiveness, elbasvir/grazoprevir, direct-acting antivirals, pegylated interferon, ribavirin, hepatitis C virus

## Abstract

**Objective:**

This study aims to systematically review recent economic evaluations of elbasvir/grazoprevir (EBR/GZR) for chronic hepatitis C (CHC), to critically appraise the reporting quality and to summarize the results.

**Methods:**

A literature search was undertaken using Medline, Embase, the Cochrane Library, EconLit, China National Knowledge Infrastructure, Wanfang Data, and Chongqing VIP to identify original articles containing economic evaluations of EBR/GZR for CHC published between 1 January 2000 and 31 December 2020. The Consolidated Health Economic Evaluation Reporting Standards statement was used to assess the quality of reporting of the articles.

**Results:**

Of 93 articles identified, 13 studies fulfilled the inclusion criteria. These studies were conducted in 4 countries, and 8 active interventions were assessed. The target population was patients infected with CHC genotype 1 infection in all studies. Eight out of 13 studies that compared EBR/GZR vs. other direct antiviral agents suggested that EBR/GZR was generally more cost-effective or dominant than daclatasvir/asunaprevir (DCV/ASV), sofosbuvir/velpatasvir (SOF/VEL), ledipasvir/sofosbuvir (LDV/SOF), ombitasvir/paritaprevir/ritonavir + dasabuvir (3D) but not more cost-effective than glecaprevir/pibrentasvir (GLE/PIB). Two studies from China and one study from the USA that compared EBR/GZR vs. pegylated interferon and ribavirin (PegIFN/RBV) consistently indicated that EBR/GZR was generally more cost-effective than PegIFN/RBV. One study from Italy compared EBR/GZR with SOF + PegIFN/RBV and suggested that EBR/GZR had a lower cost and higher effectiveness. One study from France and one study from the USA confirmed that compared with non-therapy for patients with chronic kidney disease, EBR/GZR was cost-effective at commonly accepted current standards. All included studies were of good quality of reporting, with an average score of 21.9 (range 19–23).

**Conclusion:**

EBR/GZR for CHC genotype 1 might be cost-effective or dominant compared with PegIFN/RBV and other direct antiviral agents (SOF/VEL, 3D, DCV/ASV, LDF/SOF) or non-therapy. However, under certain assumptions, EBR/GZR was not a cost-effective alternative for CHC patients vs. GLE/PIB.

## Introduction

Chronic hepatitis C (CHC) is caused by hepatitis C virus (HCV), characterized by liver damage and extreme risks of progressing liver cirrhosis or hepatocellular carcinoma (HCC) in the end stage of infection (1). According to World Health Organization (WHO) reports, ~71 million people are chronically infected with HCV, and ~399,000 people die of cirrhosis or HCC caused by HCV infection each year (2). From 2004 to 2016, a total of 2.021 million cases of CHC were reported in China, with the overall incidence increasing by 14.4% annually (3). The influencing factors of CHC disease include sex, age, lifestyle, and genotype. For instance, the most widespread genotype (GT) is HCV GT1b (62.78%) in the Chinese population, while GT1 dominates (75.3%), with GT1a being the most prevalent subtype in the USA (4–6). CHC patients suffer tremendously both physically and mentally and increase their economic burden through higher healthcare expenses. The average annual direct economic burden of CHC patients is ¥ 69,280, and the average annual direct economic burden of patients with liver cirrhosis or liver cancer is ¥ 44,750 and ¥ 101,389, respectively (7, 8). It is apparent that chronic HCV infection can lead to enormous clinical and economic burdens.

Achievement of sustained virologic response (SVR) is the endpoint criterion of treating HCV infection, which can significantly decrease the risk of liver disease progression and avoid transmutation into end-stage liver diseases (9). The treatment of HCV infection and achievement of SVR are of critical importance in decreasing the medical and economic burdens among CHC patients.

Historically, the standard of care for HCV infection is the combination of pegylated interferon (PegIFN) and ribavirin (RBV), which is usually related to low efficacy and many adverse event rates, especially in cirrhotic patients. Recently, direct-acting antivirals (DAAs) have developed rapidly with improved SVR rates and fewer adverse events in the global market (10, 11). These all-oral DAA regimens for HCV infection included elbasvir/grazoprevir (EBR/GZR), daclatasvir/asunaprevir (DCV/ASV), sofosbuvir/velpatasvir (SOF/VEL), ombitasvir/paritaprevir/ritonavir + dasabuvir (3D), glecaprevir/pibrentasvir (GLE/PIB), and ledipasvir/sofosbuvir (LDV/SOF). As more novel DAAs for HCV infection have been approved to enter the market, it is essential to evaluate the effects on both health outcomes and economics (12).

EBR/GZR is a fixed-dose DAA compound tablet with improved efficacy, safety, and higher cost that is suitable for the treatment of CHC patients with GT1 and GT4 (13). It was officially approved to enter the Chinese market in November 2019 and introduced as a new treatment regimen for chronic HCV infection in China. Since EBR/GZR entered the market, more and more studies focused on its efficacy, safety, and cost-effectiveness, which have provided massive evidence for a deeper understanding. Currently, there are few systematic reviews on EBR/GZR, and it is necessary to estimate the economic effects of these drugs in chronic HCV infection. In summary, this paper reviews and appraises the economic evidence of treatments with EBR/GZR in CHC patients. The results would provide valuable information to researchers in designing and conducting high-quality economic evaluations and to administrators as well as health workers in making best decisions.

## Methods

A systematic literature search was undertaken to identify the cost-effectiveness analyses of drugs for EBR/GZR according to the PRISMA (Preferred Reporting Items for Systematic Reviews and Meta-Analyses) guidelines followed for review and reporting procedures.

### Eligibility Criteria

Articles included in the systematic review met the following criteria: (1) the full economic evaluation, examined costs with their consequences, and reported incremental cost-effectiveness ratios (ICERs) or incremental cost-utility ratios (ICURs) were identified; (2) regardless of monotherapy or combination treatment, elbasvir/grazoprevir intervention was included; (3) complete full-text formats were available.Systematic reviews, methodological articles, expert opinions, comments (commentary), conference abstracts, and proceedings were excluded.

### Literature Search

We restricted the analysis to papers published between 1 January 2000 and 31 December 2020 about relevant studies estimating the cost-effectiveness of CHC treatments. The following databases were searched: Medline, Embase, the Cochrane Library, and EconLit databases for English-language studies and China National Knowledge Infrastructure (CNKI), Wanfang Data and Chongqing VIP (CQVIP) for Chinese-language studies. Literature search algorithm was shown in Supplementary Table 1.

### Study Selection

Two independent reviewers screened the titles and abstracts of all articles for eligibility. Full-text formats of all potentially relevant publications were obtained and reviewed to decide whether they met the pre-specified inclusion criteria. To resolve discrepancies, another discussion could be conducted.

### Data Collection

A standardized data extraction form was used to collect relevant information, such as basic information for articles (e.g., authors and publication year), characteristics of studies (e.g., design and sample size), types of economic evaluation, study objective, descriptions of the intervention and comparators, measure of benefit, cost data and respective sources, approaches for dealing with uncertainty as well as cost, and outcome results.

### Quality Assessment

All included studies were appraised for quality of reporting using the 24-item Consolidated Health Economic Evaluation Reporting Standards (CHEERS) statement (14). Each item in the CHEERS checklist was scored as having met the criteria in full (“1”), not at all (“0”), or not applicable (NA). Two reviewers independently appraised the studies, and the other authors solved the disagreements through discussion and consensus. Studies with scores higher than 75% were categorized as good, studies with scores in the range of 50–74% were categorized as moderate, and studies with scores lower than 50% were categorized as low.

### Data Synthesis

To summarize and evaluate the aims, methods, settings, and results of the studies reviewed, a narrative synthesis was used. If possible, information was compared across studies about the modeling technique, the cost perspective, the measures of benefit used, and incremental cost-effectiveness ratios. Cost/charge data are presented in US$ for the common price year 2020 using the “CCEMG-EPPI-Center Cost Converter” Version 1.6 (15), a web-based tool that can be used to adjust an estimate of cost expressed in one currency and price year to a target currency and/or price year.

## Result

### Studies Identified

**Figure 1:**
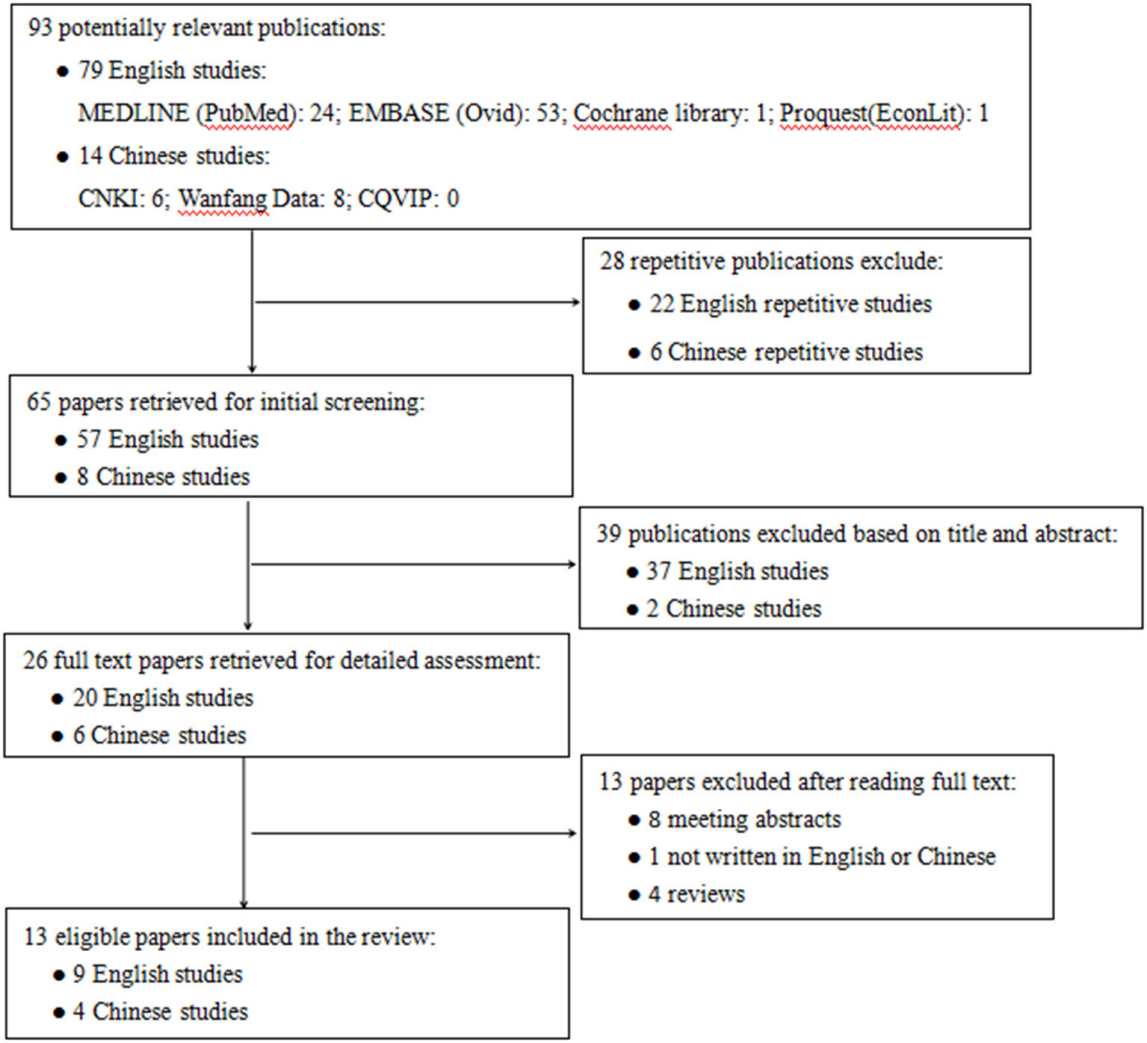
Flowchart of literature search. CNKI, China National Knowledge Infrastructure database; CQVIP, Chongqing VIP database.

Figure 1 shows the flow chart for the identification of studies. The initial database search identified 93 potentially relevant publications, of which 79 were in English language and 14 were in Chinese. Among them, 28 were excluded for repetitive publications. After the papers' initial screening, 39 publications were excluded based on title and abstract. The remaining 26 full text papers were retrieved for detailed assessment, and 13 publications were excluded for reasons such as meeting abstracts (*n* = 8), not written in English or Chinese (*n* = 1), and review articles (*n* = 4). A total of 13 publications were retrieved and analyzed for further data extraction and quality assessment.

### Description of Identified Studies

The general characteristics of the included studies are reported in Table 1. China accounted for the largest number (*n* = 7), and the remaining studies were conducted in the USA (*n* = 3), Italy (*n* = 1), France (*n* = 1), and Japan (*n* = 1). The Markov model were used in all of the studies. Seven studies were conducted from the healthcare perspective, and three were conducted from the payer perspective, while the perspective was not stated in two studies. Used in Markov models, most time horizons were applied for a lifetime, except one applied 60-year time horizon.

**Table 1:** General characteristics of the included studies.

**References**	**Year published, country**	**Perspective**	**Model type**	**Target population**	**Treatment**	**Comparator**	**Cost components**	**Time horizon**	**Discount rate (%)**	**Source of effectiveness and safety data**
Chen and Ma (16)	2020, China	Unclear	Markov model	Adult CHC patients with HCV genetype 1b	EBR/GZR	3D	Outpatient, hospitalization, medication and laboratory examination	Lifetime	5	Phase III clinical trial and meta analysis
Chen et al. (17)	2020, China	Medical and health system	Markov model	Newly treated CHC patients with HCV genetype 1b (no cirrhosis)	GLE/PIB	EBR/GZR	Expenses for diagnosis and treatment, inspections and tests, medicines and hospitalization	Lifetime	5	Phase III clinical trial
Chen et al. (18)	2019, China	Medical and health system	Markov model	Adult CHC patients with HCV genetype 1	EBR/GZR	SOF/VEL	Medicines, costs related to health status	Lifetime	5	Retrospective study and real world research
Chen et al. (19)	2018, China	Medical service provider	Markov model	Adult CHC patients with HCV genetype 1b	EBR/GZR	PegIFN/RBV	Laboratory examination, instrument examination, medication and hospitalization	Lifetime	5	Phase III clinical trial and real world research
Yuen et al. (20)	2020, Hong Kong, China	Medicare payer	Markov model	CHC patients with HCV genetype 1	EBR/GZR	PegIFN/RBV	Medicines, costs related to health status	Lifetime	3	Multi-center Phase III clinical trial
Yun et al. (21)	2020, China	Medical institution perspective	Markov model	Newly treated CHC patients with HCV genetype 1b	SOF/VEL	EBR/GZR	Medicines, costs related to health status	Lifetime	5	Multi-center Phase III clinical trial
Kawaguchi et al. (22)	2020, Japan	Public Relations and Health System	Markov model	Newly treated CHC patients with HCV genetype 1 (no cirrhosis)	GLE/PIB	EBR/GZR	Medicines, costs related to health status	Lifetime	2	Phase III clinical trial
Chen et al. (23)	2018, China	Medical and health system	Markov model	Adult CHC patients with HCV genetype 1b	EBR/GZR	DCV/ASV	Medicines, costs related to health status	Lifetime	5	Multi-center Phase III clinical trial and meta analysis
Maunoury et al. (24)	2018, France	Payer perspective	Markov model	CHC patients with HCV genetype 1, Chronic kidney disease IV-V	EBR/GZR	With no treatment	Outpatient, hospitalization, drug treatment and kidney and liver treatment costs	Lifetime	4	Multi-center Phase III clinical trial
Rolli et al. (25)	2018, Italy	National Health System	Markov model	CHC patients with HCV genetype 1	EBR/GZR	SOF+PegIFN/RBV	Medicines, costs related to health status	60 years	3	Phase III clinical trial
Corman et al. (26)	2017, USA	Payer perspective	Markov model	CHC patients with HCV genetype 1	EBR/GZR	LDV/SOF, 3D ± RBV, SOF/VEL	Drug, health status related costs, laboratory testing	Lifetime	3	Phase III clinical trial and meta analysis
Elbasha et al. (27)	2017, USA	Payer perspective	Markov model	CHC patients with HCV genetype 1, and Chronic kidney disease	EBR/GZR	With no treatment, PegIFN/RBV	Outpatient, hospitalization, drug treatment and kidney and liver treatment costs	Lifetime	unclear	Phase III clinical trial and meta analysis
Elbasha et al. (28)	2017, USA	Unclear	Markov model	CHC patients with HCV genetype 1a	EBR/GZR with RAVs	EBR/GZR without RAVs, 3D, LDV/SOF with 8 weeks, LDV/SOF with 12 weeks	Drug, health status related costs, instrument testing	Lifetime	3	Phase III clinical trial and meta analysis

*HCV, hepatitis C virus; CHC, Chronic hepatitis C; EBR/GZR, elbasvir/grazoprevir; 3D, ombitasvir/paritaprevir/ritonavir+dasabuvir; GLE/PIB, glecaprevir/pibrentasvir; SOF/VEL, sofosbuvir/velpatasvir; PegIFN/RBV, Pegylated interferon/ribavirin; DCV/ASV, daclatasvir/asunaprevir; SOF, sofosbuvir; LDV/SOF, ledipasvir/sofosbuvir; RBV, ribavirin; RAVs, resistance-associated variants*.

The direct antiviral agents (DAAS) for CHC in the included studies were EBR/GZR, GLE/PIB, SOF/VEL, and DCV/ASV, and the most frequent applications for comparators were PegIFN/RBV and non-therapy. Three studies were funded by Merck & Co., Inc., one by Merck Sharp & Dohme Corp., Inc., one by Gilead Sciences Shanghai Pharmaceutical Technology Co., Ltd., one by AbbVie Inc. and AbbVie GK, one by MSD Italia S.r.l., and one by National Natural Science Foundation of China. The remaining studies did not indicate the source of funding or were without funding.

### Quality Assessment

The results of reporting quality per study as assessed by the CHEERS statement are summarized in Supplementary Table 2. In the CHEERS checklist, each item reported sufficiently, partially, or not at all in the review was declared. All of the included studies were evaluated for high quality. Two studies did not state the perspective (16, 28), and only one study did not report the discount rate. Meanwhile, the currency, price date, and conversion were not stated in four studies (16–19). Although some of the study findings and limitations were reported, none of the studies stated the generalizability of the results. The source of funding was not reported in four studies (16, 18, 19, 24), and four studies failed to report conflicts of interest.

### Cost-Effectiveness Results of the Studies

The results of the overview of the economic evaluation outcomes of the included studies are summarized in Supplementary Table 3.

#### Elbasvir/Grazoprevir vs. Other Direct Anti-virus Agents

Eight studies provided economic evaluation for EBR/GZR vs. other DAAs. Five of them were conducted in China, and four studies suggested that EBR/GZR was more cost-effective than 3D (16), SOF/VEL (18, 21), and DCV/ASV (23). However, the remaining study (17) suggested that GLE/PIB has cost-effectiveness advantages over EBR/GZR in CHC GT1b treatment-naive patients without cirrhosis, which is the same as the result of a study (22) conducted in Japan. Two studies were conducted in the USA; one study (26) showed that compared with 3D, SOF/VEL, or LDV/SOF, EBR/GZR was the economically dominant regimen for treating patients with GT1a or GT1b CHC, and the other study (28) showed that resistance-associated variant (RAV) testing before treatment with EBR/GZR was more cost-effective than EBR/GZR without testing, LDV/SOF, or 3D for treating patients with GT1a CHC.

#### Elbasvir/Grazoprevir vs. PegIFN/RBV

Three studies considered PegIFN/RBV as comparators (19, 20, 27). Chen et al. (19) assumed that EBR/GZR provided more QALYs and lower costs than PegIFN/RBV for patients in China with cirrhosis or not. Yuen et al. (20) demonstrated that patients with GT1a or GT1b CHC in Hong Kong could gain more QALYs (1.3840 QALYs in GT1a, 0.8227 QALYs in GT1b) treated with EBR/GZR than PegIFN/RBV, while the costs were much higher ($ 6942 in GT1a, $ 7123 in GT1b). In total, EBR/GZR was cost-effective compared to PegIFN/RBV in GT1. The same result was as Elbasha et al. (27).

#### Elbasvir/Grazoprevir vs. PegIFN/RBV + SOF

One study (25) conducted in Italy compared EBR/GZR with SOF + PegIFN/RBV and suggested that the EBR/GZR group has a lower cost and higher effectiveness, which has an absolute advantage.

#### Elbasvir/Grazoprevir vs. Non-therapy

Two studies (24, 27) conducted in France and the USA compared EBR/GZR with non-therapy. Maunoury et al. (24) indicated that EBR/GZR compared to no treatment was considered cost-effective for patients with renal insufficiency at a willingness to pay of €20,000/QALY. Additionally, Elbasha et al. (27) showed that EBR/GZR resulted in higher average remaining QALYs and higher costs compared with non-therapy for patients with chronic kidney disease, and the ICER was $13201.34/QALY, which was less than the thresholds of $100,000/QALY. EBR/GZR is considered cost-effective at the commonly accepted current U.S. standards.

## Discussion

### Summary of Evidence

Thirteen economic evaluations of drugs for chronic hepatitis were identified in our systematic review from 2000 to 2020. Nine of them were written in English, and the other was written in Chinese. Eight active interventions were assessed in this research, including EBR/GZR, GLE/PIB, SOF/VEL, PegIFN/RBV, 3D, DCV/ASV, LDF/SOF, and PegIFN/RBV + SOF. This study aimed to evaluate the costs and cost-effectiveness of EBR/GZR for chronic hepatitis. If applicable, the thresholds were stated in the included studies. Meanwhile, we found the proper and accepted thresholds used in corresponding countries to evaluate whether the ICERs of EBR/GZR were below these thresholds. According to the results, decisions were made to determine whether they were valuable or cost-effective for CHC.

When compared to PegIFN/RBV, EBR/GZR was cost-effective or dominant for CHC patients with GT1a or GT1b or not in the included studies (19, 20, 27). Three studies assessed the cost-effectiveness analysis of EBR/GZR vs. SOF/VEL, suggesting that EBR/GZR was cost-effective in general but not for CHC patients with GT1b TN non-cirrhosis (18, 21, 26). According to the results of two studies, GLE/PIB might be a more cost-effective front-line therapy than EBR/GZR due to its economic advantages (17, 22). Two other studies investigated whether EBR/GZR was more cost-effective or dominant than 3D (26, 28).

### Quality of Evidence

To appraise the quality of the included studies in our reviews, the availability of the CHEERS statement can be used to improve and hence the quality of economic evaluations of CHC. However, we find that some studies are of insufficient quality.

In terms of characterizing the study findings, limitations, generalizability, and current knowledge, it is necessary for readers to quickly obtain information on limitations and current research status. It also helps improve the reporting of economic evaluations in the future. However, in these included studies, none of them reported information that might not be objective.

In addition, some of the studies included in our review are funded by pharmaceutical companies, which might lead to potential bias of the economical evaluations. Although it is common sense for evaluations influenced by funded companies, an appropriate approach to assess this bias remains uncertain.

### Key Elements of Cost-Effectiveness

Consistent with previous research, some key elements of cost-effectiveness were also found in our studies. On the one hand, the election of the comparator is crucial, and it could lead to different cost-effectiveness of the therapy regimen. For instance, EBR/GZR was evaluated to be dominant compared to SOF/VEL or DCV/ASV, while the cost-effectiveness was at a disadvantage compared to GLE/PIB. Therefore, the selection of the comparator is one of the most critical structures of cost-effectiveness analysis.

On the other hand, the included analyses were mainly for specific countries, so the results and final conclusions of economic assessments may be significantly affected by the healthcare systems and medical insurance policies of different countries. To enhance the universality and transferability of research across settings, future evaluations need to use improved methods, such as constructing multi-level models to analyze cost-effectiveness data and identifying a series of appropriate covariates to address assumptions and uncertainties in the results of economic evaluations.

### Strengths and Limitations

So far as we know, this article is the first systematic review of published literature to assess the cost-effectiveness properties of EBR/GZR for chronic HCV infection. Unlike previous narrative and systematic studies that were antiquated or limited to a single comparator, this article is the most extensive review, including literature retrieval and selection, economic assessment over a longer period of time, and the use of validated tools to assess quality. Additionally, this review includes all published cost-effectiveness studies on EBR/GZR, and all cost-related parameters of different regional backgrounds are adjusted to 2020 dollars for more convenient comparison.

Although this review uses scientific and systematic methods to minimize deviations, several limitations that influence the conclusions should be considered when explicating the results.

First, in view of the possible divergences in the selected literature backgrounds and economic evaluation methods, it is exceedingly difficult to integrate these studies into a coherent whole. Although the included literature adopts the Markov model when analyzing cost-effectiveness, there are still diversified differences between them, such as research perspectives, time horizons, payers, target populations, and healthcare systems. Thus, we summarized the evidence qualitatively and then cautiously interpreted the results and conclusions.

Second, most of the literature included in this study is from the perspectives of the payer or the healthcare system and only considers direct medical costs while overlooking indirect costs. If chronic HCV infection is not treated in time, it will cause tremendous productivity and economic losses to society. Therefore, further research on pharmaceutical economics from the perspective of the whole society needs to be carried out.

Third, since some relevant studies with negative results or unfavorable findings may not be published, the literature search results are biased to a certain extent in our review. Meanwhile, due to the limitation of the author's language, some related potential documents may be ignored, especially in languages other than Chinese and English.

## Conclusions

In conclusion, 13 studies were included on the cost-effectiveness of drugs for chronic hepatitis in this review. According to the available evidence, EBR/GZR for CHC might be cost-effective or dominant compared with PegIFN/RBV and other DAAs (SOF/VEL, 3D, DCV/ASV, LDF/SOF) or non-therapy. However, under certain assumptions, EBR/GZR was not a cost-effective alternative for CHC patients with cirrhosis or not or vs. GLE/PIB. More attention should be taken to improve the quality of reporting of economic evaluations.

## Data Availability Statement

The original contributions presented in the study are included in the article/Supplementary Materials, further inquiries can be directed to the corresponding author/s.

## Author Contributions

RY and JL: study design and study conduct. JL and MG: data collection. JL and LK: data analysis and drafting manuscript. RY, LK, MG, and JL: data interpretation. All authors contributed to the article and approved the submitted version.

## Conflict of Interest

The authors declare that the research was conducted in the absence of any commercial or financial relationships that could be construed as a potential conflict of interest.

## Publisher's Note

All claims expressed in this article are solely those of the authors and do not necessarily represent those of their affiliated organizations, or those of the publisher, the editors and the reviewers. Any product that may be evaluated in this article, or claim that may be made by its manufacturer, is not guaranteed or endorsed by the publisher.
