# Supplementary material for: Cost-Effectiveness of Elbasvir/Grazoprevir for the Treatment of Chronic Hepatitis C: A Systematic Review
Source: Front Public Health. 2022 May 13;10:836986. doi: 10.3389/fpubh.2022.836986 (PMC9136222; doi:10.3389/fpubh.2022.836986)
Supplement: Supplementary file 1 [file Table_1.docx]

Table S1. Literature search algorithm

**MEDLINE (PubMed)**

| Search strategy | | Item found |
| --- | --- | --- |
| #1 | (Cost-benefit analysis[MeSH] OR (cost*[tiab] AND (benefit[tiab] OR effectiveness[tiab] OR utility[tiab]) OR (marginal[tiab] AND analys*[tiab]) OR minimization OR minimisation) OR ((economic*[tiab] OR pharmacoeconomic*[tiab]) AND (analys*[tiab] OR assessment*[tiab] OR evaluat*[tiab] OR implication[tiab] OR health[tiab]))) | 836916 |
| #2 | Hepatitis C[MeSH] OR Hepacivirus[MeSH] OR Hepatitis C Antibodies[MeSH] OR Hepatitis C Antigens[Mesh] OR Hepatitis C[tiab] OR hepaciviru*[tiab] OR hcv[tiab] OR hep c[tiab] | 99125 |
| #3 | (((grazoprevir[tiab]) OR (MK5172[tiab])) AND ((elbasvir[tiab]) OR (MK8742[tiab]))) OR (ZEPATIER[tiab]) | 293 |
| #4 | #1 AND #2 AND #3 AND ("2000/01/01"[Date - Publication] : "2020/12/31"[Date - Publication]) | 24 |

**EMBASE (Ovid)**

| #1 | (‘Cost benefit analysis’/exp OR (cost*:ti,ab,kw AND (benefit:ti,ab,kw OR effectiveness:ti,ab,kw OR utility:ti,ab,kw) OR (marginal:ti,ab,kw AND analys*:ti,ab,kw) OR minimization:ti,ab,kw OR minimisation:ti,ab,kw) OR ((economic*:ti,ab,kw OR pharmacoeconomic*:ti,ab,kw) AND (analys*:ti,ab,kw OR assessment*:ti,ab,kw OR evaluat*:ti,a,kw OR implication:ti,ab,kw OR health:ti,ab,kw))) | 529537 |
| --- | --- | --- |
| #2 | ((‘Hepatitis C’/exp OR ‘Hepacivirus’/ exp OR ‘Hepatitis C Antibodies’/exp OR ‘Hepatitis C Antigens’/exp) OR (‘Hepatitis C’:ti,ab,kw OR ‘hepaciviru*’:ti,ab,kw OR ‘hcv’:ti,ab,kw OR ‘hep c’:ti,ab,kw) | 217064 |
| #3 | ((grazoprevir:ti,ab,kw OR MK5172:ti,ab,kw) AND (elbasvir:ti,ab,kw OR MK8742 :ti,ab,kw) ) OR (ZEPATIER:ti,ab,kw) | 945 |
| #4 | #1 AND #2 AND #3AND ([2000-2020]/py) | 53 |

**Cochrane library**

| #1 | (MeSH descriptor: [Cost benefit analysis] explode all trees OR (cost*:ti,ab,kw AND (benefit OR effectiveness OR utility OR minimization OR minimisation):ti,ab,kw OR (marginal AND analys*):ti,ab,kw) OR ((economic* OR pharmacoeconomic*):ti,ab,kw AND (analys* OR assessment* OR evaluat* OR implication OR health):ti,ab,kw)) | 40348 |
| --- | --- | --- |
| #2 | ((MeSH descriptor: [Hepatitis C] explode all trees OR MeSH descriptor: [Hepacivirus] explode all trees OR MeSH descriptor: [Hepatitis C Antibodies] explode all trees OR MeSH descriptor: [Hepatitis C Antigens] explode all trees) OR (‘Hepatitis C’ OR ‘hepaciviru*’ OR ‘hcv’ OR ‘hep c’) :ti,ab,kw. | 13477 |
| #3 | (grazoprevir OR MK5172):ti,ab,kw AND (elbasvir OR MK8742):ti,ab,kw OR (ZEPATIER):ti,ab,kw | 157 |
| #4 | #1 AND #2 AND #3AND ([2000-2020]/py) | 1 |

**Proquest(EconLit)**

| #1 | su(Cost benefit analysis) OR ( (TI,AB(cost*) AND TI,AB(benefit OR effectiveness OR utility) OR TI,AB (marginal AND analys*) OR TI,AB (minimization OR minimisation))OR TI,AB(analys* OR assessment*OR evaluat* OR implication OR health) AND TI,AB(economic* OR pharmacoeconomic*)) | 238118 |
| --- | --- | --- |
| #2 | su((Hepatitis C) OR (Hepacivirus) OR (Hepatitis C Antibodies) OR (Hepatitis C Antigens))OR TI,AB(‘Hepatitis C’ OR ‘hepaciviru*’ OR ‘hcv’ OR ‘hep c’) | 23505 |
| #3 | TI, AB(grazoprevir OR MK5172) AND TI, AB(elbasvir OR MK8742 ) OR TI, AB(ZEPATIER) | 3 |
| #4 | #1 AND #2 AND #3AND ([2000-2020]/py) | 1 |

**Chinese database: China National Knowledge Infrastructure (CNKI)**

| Search strategy | Item found |
| --- | --- |
| (TI&KY&AB=丙型肝炎 OR TI&KY&AB=丙肝) AND (TI&KY&AB=费用 OR TI&KY&AB=成本 OR TI&KY&AB=经济 OR TI&KY&AB=负担 OR TI&KY&AB=卫生支出 OR TI&KY&AB=卫生费用) AND (TI&KY&AB=艾尔巴韦OR TI&KY&AB=格拉瑞韦)  Publication Date : 2000-01-01 to 2021-12-31 | 6 |

**Chinese database: Wanfang Data**

| Search strategy | Item found |
| --- | --- |
| [全部字段=(丙型肝炎 OR 丙肝) AND 全部字段=(费用OR 成本OR 经济 OR 负担 OR 卫生支出 OR 卫生费用) AND 全部字段=(艾尔巴韦 OR 格拉瑞韦)](http://med.wanfangdata.com.cn/Paper/Search?q=((((%E4%B8%99%E5%9E%8B%E8%82%9D%E7%82%8E)%20OR%20%E4%B8%99%E8%82%9D))%20AND%20((((((%E8%B4%B9%E7%94%A8)%20OR%20%E6%88%90%E6%9C%AC)%20OR%20%E7%BB%8F%E6%B5%8E)%20OR%20%E8%B4%9F%E6%8B%85)%20OR%20%E5%8D%AB%E7%94%9F%E6%94%AF%E5%87%BA)))%20AND%20(((%E8%89%BE%E5%B0%94%E5%B7%B4%E9%9F%A6)%20AND%20%E6%A0%BC%E6%8B%89%E7%91%9E%E9%9F%A6)))  Publication Date : 2000 to 2020 | 8 |

**Chinese database: the Chongqing VIP (CQVIP)**

| Search strategy | Item found |
| --- | --- |
| M=(丙型肝炎 OR 丙肝) AND M=(费用 OR 成本 OR 经济 OR 负担 OR 卫生支出 OR 卫生费用) AND M=(艾尔巴韦 OR 格拉瑞韦)  Publication Date : 2000 to 2020 | 0 |
