# Supplementary material for: Cost-Effectiveness of Elbasvir/Grazoprevir for the Treatment of Chronic Hepatitis C: A Systematic Review
Source: Front Public Health. 2022 May 13;10:836986. doi: 10.3389/fpubh.2022.836986 (PMC9136222; doi:10.3389/fpubh.2022.836986)
Supplement: Supplementary file 2 [file Table_2.docx]

Table S2: Quality of the economic evaluations (as assessed by the CHEERS statement)

| Item No. | Section/item | Chen et al., 2020a | Chen et al., 2020b | Chen et al., 2019 | Chen et al., 2018a | Yuen et al., 2020 | Yun et al., 2020 | Kawaguchi ea al., 2020 | Chen et al., 2018b | Maunoury et al., 2018 | Rolli el al., 2018 | Corman et al., 2017 | Elbasha et al., 2017a | Elbasha et al., 2017b |
| --- | --- | --- | --- | --- | --- | --- | --- | --- | --- | --- | --- | --- | --- | --- |
| 1 | Title | 1 | 1 | 1 | 1 | 1 | 1 | 1 | 1 | 1 | 1 | 1 | 1 | 1 |
| 2 | Abstract | 1 | 1 | 1 | 1 | 1 | 1 | 1 | 1 | 1 | 1 | 1 | 1 | 1 |
| 3 | Background and  objectives | 1 | 1 | 1 | 1 | 1 | 1 | 1 | 1 | 1 | 1 | 1 | 1 | 1 |
| 4 | Target population  And subgroups | 1 | 1 | 1 | 1 | 1 | 1 | 1 | 1 | 1 | 1 | 1 | 1 | 1 |
| 5 | Setting and  location | 1 | 1 | 1 | 1 | 1 | 1 | 1 | 1 | 1 | 1 | 1 | 1 | 1 |
| 6 | Study perspective | 0 | 1 | 1 | 1 | 1 | 1 | 1 | 1 | 1 | 1 | 1 | 1 | 0 |
| 7 | Comparators | 1 | 1 | 1 | 1 | 1 | 1 | 1 | 1 | 1 | 1 | 1 | 1 | 1 |
| 8 | Time horizon | 1 | 1 | 1 | 1 | 1 | 1 | 1 | 1 | 1 | 1 | 1 | 1 | 1 |
| 9 | Discount rate | 1 | 1 | 1 | 1 | 1 | 1 | 1 | 1 | 1 | 1 | 1 | 0 | 1 |
| 10 | Choice of health  outcomes | 1 | 1 | 1 | 1 | 1 | 1 | 1 | 1 | 1 | 1 | 1 | 1 | 1 |
| 11 | Measurement of  effectiveness | 1 | 1 | 1 | 1 | 1 | 1 | 1 | 1 | 1 | 1 | 1 | 1 | 1 |
| 12 | Measurement and  Valuation of preference based outcomes | 1 | 1 | 1 | 1 | 1 | 1 | 1 | 1 | 1 | 1 | 1 | 1 | 1 |
| 13 | Estimating resources  and costs | 1 | 1 | 1 | 1 | 1 | 1 | 1 | 1 | 1 | 1 | 1 | 1 | 1 |
| 14 | Currency, price date, and  conversion | 0 | 0 | 0 | 0 | 1 | 1 | 1 | 1 | 1 | 1 | 1 | 1 | 1 |
| 15 | Choice of model | 1 | 1 | 1 | 1 | 1 | 1 | 1 | 1 | 1 | 1 | 1 | 1 | 1 |
| 16 | Assumptions | 1 | 1 | 1 | 1 | 1 | 1 | 1 | 1 | 1 | 1 | 1 | 1 | 1 |
| 17 | Analytical methods | 1 | 1 | 1 | 1 | 1 | 1 | 1 | 1 | 1 | 1 | 1 | 1 | 1 |
| 18 | Study parameters | 1 | 1 | 1 | 1 | 1 | 1 | 1 | 1 | 1 | 1 | 1 | 1 | 1 |
| 19 | Incremental costs and outcomes | 1 | 1 | 1 | 1 | 1 | 1 | 1 | 1 | 1 | 1 | 1 | 1 | 1 |
| 20 | Characterizing uncertainty | 1 | 1 | 1 | 1 | 1 | 1 | 1 | 1 | 1 | 1 | 1 | 1 | 1 |
| 21 | Characterizing heterogeneity | 1 | 1 | 1 | 1 | 1 | 1 | 1 | 1 | 1 | 1 | 1 | 1 | 1 |
| 22 | Study findings, limitations,  generalizability, and current  knowledge | 0 | 0 | 0 | 0 | 0 | 0 | 0 | 0 | 0 | 0 | 0 | 0 | 0 |
| 23 | Source of funding | 0 | 1 | 0 | 0 | 1 | 1 | 1 | 1 | 1 | 1 | 1 | 1 | 1 |
| 24 | Conflicts of interest | 0 | 0 | 0 | 0 | 1 | 1 | 1 | 1 | 1 | 1 | 1 | 1 | 1 |
| Overall quality | | Good | Good | Good | Good | Good | Good | Good | Good | Good | Good | Good | Good | Good |

Note:“1” meets the quality assessment criteria; “0” does not fully conform to the quality assessment criteria; CHEERS, Consolidated Health Economic Evaluation Reporting Standards.
