# Supplementary material for: Cost-Effectiveness of Elbasvir/Grazoprevir for the Treatment of Chronic Hepatitis C: A Systematic Review
Source: Front Public Health. 2022 May 13;10:836986. doi: 10.3389/fpubh.2022.836986 (PMC9136222; doi:10.3389/fpubh.2022.836986)
Supplement: Supplementary file 3 [file Table_3.docx]

Table S3: Overview of economic evaluation outcomes of included studies

| **References** | **Comparators** | **Effectiveness** | **Costs (original currency; mean)** | **Costs (2020 US$; mean)** | **ICER (2020 US$**  **per QALY)** | **Threshold of ICER (2020 US$ per QALY)** | **Uncertainty**  **analysis** |
| --- | --- | --- | --- | --- | --- | --- | --- |
| Chen et al., 2020a | EBR/GZR vs. 3D | Incremental effectiveness (noncirrhotic): 0.0475 QALYs (Treatment 13.8675 QALYs, Comparator 13.8200 QALYs)  Incremental effectiveness (cirrhosis): 0.0178 QALYs (Treatment 12.8416 QALYs, Comparator 12.8238 QALYs) | Incremental costs (noncirrhotic): ¥ -190.73 (Treatment ¥82090.82, Comparator ¥82281.55)  Incremental costs (cirrhotic): ¥912 (Treatment ¥225807.703, Comparator¥ 224895.70) | Incremental costs (noncirrhotic):US$ -56.47 (Treatment US$24302.15, Comparator US$24358.62)  Incremental costs (cirrhotic): US$269.99 (Treatment US$66848.08, Comparator US$ 66578.09) | Patients with noncirrhotic: Compared with the control group, the treatment group has lower cost and higher effectiveness, and ICER is negative, which has an absolute advantage.  Patients with cirrhotic: US$15167.98/QALY | US$50088.08/QALY | The drug prices and SVR rates of the two treatment options have an impact on the results. |
| Chen et al., 2020b | GLE/PIB vs. EBR/GZR | Incremental effectiveness: 0.07 QALYs (Treatment 14.97 QALYs, Comparator 14.90 QALYs) | Incremental costs: ¥6462 (Treatment ¥68800, Comparator ¥62338) | Incremental costs: US$1878.12 (Treatment US$19996.09, Comparator US$18117.97) | US$26830.29.94/QALY | US$61812.33/QALY | The SVR rate and research time limit of gorcarevir/pirentavir and elbavir/glarevir have a greater impact on the results. |
| Chen et al., 2019 | EBR/GZR vs. SOF/VEL | Incremental effectiveness (noncirrhotic): 0.0613 QALYs (Treatment 13.8442 QALYs, Comparator 13.7892 QALYs)  Incremental effectiveness (cirrhosis): 0.1117 QALYs (Treatment 12.6687 QALYs, Comparator 12.5578 QALYs) | Incremental costs (noncirrhotic): ¥-12270.64 (Treatment ¥82199.80, Comparator ¥94470.40)  Incremental costs (cirrhotic): ¥ -12758.10 (Treatment ¥230026.40, Comparator¥242784.50) | Incremental costs (noncirrhotic): US$-3632.58 (Treatment US$24334.42, Comparator US$27967.00)  Incremental costs (cirrhotic): US$ -3776.91 (Treatment US$68096.98, Comparator US$71873.89) | Regardless of whether it is combined with liver cirrhosis, the treatment group has lower cost and higher effectiveness than the control group. ICER is negative and has an absolute advantage. | US$53287.18/QALY | When the SVR of both regimens, the recurrence rate of EBR/GZR and unit price are changed within the set range, the research conclusions will not be changed.  When the price of the SOF/VEL program takes the lowest value or the recurrence rate uses the lower limit, it will influence the results of basic research. |
| Chen et al., 2018a | EBR/GZR vs. PegIFN/RBV | Incremental effectiveness (noncirrhotic): 1.1713 QALYs (Treatment 13.8675 QALYs, Comparator 12.6962 QALYs)  Incremental effectiveness (cirrhosis): 3.9492 QALYs (Treatment 12.8416 QALYs, Comparator 8.8924 QALYs) | Incremental costs (noncirrhotic): ¥-40700.73 (Treatment ¥82090.82, Comparator¥122791.55)  Incremental costs (cirrhotic): ¥ -100737.31(Treatment ¥225807.70, Comparator ¥326545.01) | Incremental costs (noncirrhotic): US$-12599.25 (Treatment US$25411.89, Comparator US$38011.14)  Incremental costs (cirrhotic): US$ -31184.07 (Treatment US$ 69900.65, Comparator US$101084.72) | Compared with the control group, the treatment group has lower cost and higher effectiveness, and ICER is negative, which has an absolute advantage. | US$50088.08/QALY | Results robust |
| Yuen et al., 2020 | EBR/GZR vs. PegIFN/RBV | Incremental effectiveness (genotype 1a): 1.3840 QALYs (Treatment 10.9784 QALYs, Comparator 9.5944 QALYs)  Incremental effectiveness (genotype 1b): 0.8227 QALYs (Treatment 8.8083 QALYs, Comparator 7.9855 QALYs) | Incremental costs (genotype 1a): US$ 6942 (Treatment US$ 12966, Comparator US$ 6025)  Incremental costs (genotype 1b): US$ 7123 (Treatment US$ 12660, Comparator US$ 5537) | Incremental costs (genotype 1a): US$ 7211.96 (Treatment US$ 13472.16, Comparator US$ 6260.20)  Incremental costs (genotype 1b): US$ 7401.06 (Treatment US$ 13154.21, Comparator US$ 5753.15) | Patients with genotype 1a: US$ 5210.95 / QALY  Patients with genotype 1b: US$ 8996.06 / QALY | US$ 5195.19-11429.41/QALY | Results robust |
| Yun et al., 2020 | SOF/VEL vs. EBR/GZR | Incremental effectiveness: 0.0040 QALYs (Treatment 13.4263 QALYs, Comparator 13.4263 QALYs) | Incremental costs: US$ 1436 (Treatment US$ 11402, Comparator US$ 9966) | Incremental costs: US$ 1465.99 (Treatment US$ 11640.10, Comparator US$ 10174.11) | US$ 366497.5/QALY | US$ 28692.91/QALY | When the price of SOF/VEL scheme is taken to the minimum value, the result will be affected. |
| Kawaguchi ea al., 2020 | GLE/PIB vs. EBR/GZR | Incremental effectiveness: 0.13 QALYs (Treatment 14.20 QALYs, Comparator 14.07 QALYs) | Incremental costs: ￥-865000 (Treatment ￥4131000, Comparator ￥4996000) | Incremental costs: US$-9101.88 (Treatment US$43468.06, Comparator US$52569.94) | US$-70014.46/QALY | US$52612.03/QALY | Results robust |
| Chen et al., 2018b | EBR/GZR vs. DCV/ASV | First-treated patients with genotype 1b and noncirrhotic  Incremental effectiveness: 0.3617 QALYs (Treatment 13.8924 QALYs, Comparator 13.5307 QALYs)  First-treated patients with genotype 1b and cirrhotic  Incremental effectiveness: 0.4752 QALYs (Treatment 12.5463 QALYs, Comparator 12.0711 QALYs)  Non-first treatment patients with genotype 1b and noncirrhotic  Incremental effectiveness: 0.6998 QALYs (Treatment 13.9481 QALYs, Comparator 13.2483 QALYs)  Non-first treatment patients with genotype 1b and cirrhotic  Incremental effectiveness: 1.1530 QALYs (Treatment 12.7194 QALYs, Comparator 11.5664 QALYs) | First-treated patients with genotype 1b and noncirrhotic  Incremental costs: US$ -1946.60 (Treatment US$ 11771.72, Comparator US$ 13718.32)  First-treated patients with genotype 1b and cirrhotic  Incremental costs: US$ -1521.35 (Treatment US$ 34687.26, Comparator US$ 36208.60)  Non-first treatment patients with genotype 1b and noncirrhotic  Incremental costs: US$ -3998.19 (Treatment US$11421.61, Comparator US$ 15419.80)  Non-first treatment patients with genotype 1b and cirrhotic  Incremental costs: US$ -4102.21 (Treatment US$ 34017.10, Comparator US$ 38119.31) | First-treated patients with genotype 1b and noncirrhotic  Incremental costs: US$ -2022.59 (Treatment US$ 12231.25, Comparator US$ 14253.84)  First-treated patients with genotype 1b and cirrhotic  Incremental costs: US$ -1580.73 (Treatment US$ 36041.35, Comparator US$ 37622.08)  Non-first treatment patients with genotype 1b and noncirrhotic  Incremental costs: US$ -4154.27 (Treatment US$11867.48, Comparator US$ 16021.75)  Non-first treatment patients with genotype 1b and cirrhotic  Incremental costs: US$ -4262.35 (Treatment US$ 35345.03, Comparator US$ 39607.38) | Regardless of whether it is combined with liver cirrhosis, or has undergone standard treatment in the past, the treatment group has lower cost and higher effectiveness than the control group, and ICER is negative, which has an absolute advantage. | US$26072.30/QALY | The drug prices and SVR rates of the two treatment options have an impact on the results. |
| Maunoury et al., 2018 | EBR/GZR vs. no treatment | Incremental effectiveness: 2.47 QALYs (Treatment 6.20 QALYs, Comparator 3.73 QALYs) | Incremental costs: € 37547 (Treatment € 296672, Comparator € 259125) | Incremental costs: US$ 45689.41 (Treatment US$ 361008.04, Comparator US$ 315318.63) | US$ 18497.74/QALY | US$ 24337.18/QALY | The progression risk of chronic kidney disease has the most significant impact on the results of basic research. |
| Rolli el al., 2018 | EBR/GZR vs. PegIFN/RBV+SOF | Incremental effectiveness: 431.94 QALYs (Treatment 19287.90 QALYs, Comparator 19287.90 QALYs) | Incremental costs: € -10800156.37 (Treatment € 21104253.74, Comparator € 31904410.11) | Incremental costs: US$ -12957333.95 (Treatment US$ 25319528.17, Comparator US$ 38276862.12) | Compared with the control group, the treatment group has lower cost and higher effectiveness, and ICER is negative, which has an absolute advantage. | US$ 35992.07/QALY | Results robust |
| Corman et al., 2017 | EBR/GZR vs. 3D±RBV regimen | First-treated patients with genotype 1a and noncirrhotic Incremental effectiveness: 0.0507 QALYs (Treatment 15.3308 QALYs, Comparator 15.2802 QALYs)  First-treated patients with genotype 1a and cirrhotic Incremental effectiveness: 0.2804 QALYs (Treatment 13.9260 QALYs, Comparator 13.6456 QALYs)  Non-first treatment patients with genotype 1a and noncirrhotic Incremental effectiveness: 0.0435 QALYs (Treatment 15.3308 QALYs, Comparator 15.2873 QALYs)  Non-first treatment patients with genotype 1a and cirrhotic Incremental effectiveness: 0.0055 QALYs (Treatment 13.9260 QALYs, Comparator 13.9205 QALYs)  First-treated patients with genotype 1b and noncirrhotic Incremental effectiveness: -0.0269 QALYs (Treatment 15.3343 QALYs, Comparator 15.3613 QALYs)  First-treated patients with genotype 1b and cirrhotic Incremental effectiveness: 0.2969 QALYs (Treatment 14.0203 QALYs, Comparator 13.7234 QALYs)  Non-first treatment patients with genotype 1b and noncirrhotic Incremental effectiveness: -0.0008 QALYs (Treatment 15.3771 QALYs, Comparator 15.3779 QALYs)  Non-first treatment patients with genotype 1b and cirrhotic Incremental effectiveness: -0.0003 QALYs (Treatment 14.0203 QALYs, Comparator 14.0206 QALYs) | First-treated patients with genotype 1a and noncirrhotic Incremental costs: US$ -26973 (Treatment US$ 62337, Comparator US$89310)  First-treated patients with genotype 1a and cirrhotic Incremental costs: US$ -108573 (Treatment US$ 78201, Comparator US$ 186774)  Non-first treatment patients with genotype 1a and noncirrhotic Incremental costs: US$ -26865 (Treatment US$ 62337, Comparator US$ 89202)  Non-first treatment patients with genotype 1a and cirrhotic Incremental costs: US$ -105355 (Treatment US$ 78201, Comparator US$ 183555)  First-treated patients with genotype 1b and noncirrhotic Incremental costs: US$ -27499 (Treatment US$ 59758, Comparator US$ 87257)  First-treated patients with genotype 1b and cirrhotic Incremental costs: US$ -31754 (Treatment US$ 74557, Comparator US$ 106312)  Non-first treatment patients with genotype 1b and noncirrhotic Incremental costs: US$ -27894 (Treatment US$ 59111, Comparator US$87005)  Non-first treatment patients with genotype 1b and cirrhotic Incremental costs: US$ -28275 (Treatment US$74557, Comparator US$102832) | First-treated patients with genotype 1a and noncirrhotic Incremental costs: US$ -29202.71 (Treatment US$ 67490.07, Comparator US$96692.78)  First-treated patients with genotype 1a and cirrhotic Incremental costs: US$ -117548.15 (Treatment US$ 84665.46, Comparator US$ 202213.61)  Non-first treatment patients with genotype 1a and noncirrhotic Incremental costs: US$ -29085.78 (Treatment US$ 67490.07, Comparator US$ 96575.85)  Non-first treatment patients with genotype 1a and cirrhotic Incremental costs: US$ -114063.05 (Treatment US$ 84665.46, Comparator US$ 198728.51)  First-treated patients with genotype 1b and noncirrhotic Incremental costs: US$ -29772.19 (Treatment US$ 64697.88, Comparator US$ 94470.07)  First-treated patients with genotype 1b and cirrhotic Incremental costs: US$ -34380.02 (Treatment US$ 80720.23, Comparator US$ 115100.25)  Non-first treatment patients with genotype 1b and noncirrhotic Incremental costs: US$ -30199.85 (Treatment US$ 63997.39, Comparator US$94197.24)  Non-first treatment patients with genotype 1b and cirrhotic Incremental costs: US$ -30612.34 (Treatment US$80720.23, Comparator US$111332.57) | For patients with genotype 1a, whether or not they have liver cirrhosis, or have undergone standard treatment in the past, the treatment group have lower cost and higher utility than the control group. ICERs are negative, which have an absolute advantage.  For genotype 1b, patients with first-treated noncirrhosis, non-first treatment noncirrhosis, and non-first treatment cirrhosis, the ICERs are US$ 1106.772.86/QALY, US$ 37749812.50/QALY and US$ 102041133.33/QALY, respectively.  For first-treated patients with genotype 1b and cirrhotic, the treatment group has lower cost and higher effectiveness than the control group. ICER is negative, which has an absolute advantage. | US$ 204752.27/QALY | The drug prices and SVR rates of the two treatment options have an impact on the results. |
|  | EBR/GZR vs. LDV/SOF | First-treated patients with genotype 1a and noncirrhotic Incremental effectiveness: 0.0214 QALYs (Treatment 15.3308 QALYs, Comparator 15.3094 QALYs)  First-treated patients with genotype 1a and cirrhotic Incremental effectiveness: 0.0595 QALYs (Treatment 13.9260 QALYs, Comparator 13.8665 QALYs)  Non-first treatment patients with genotype 1a and noncirrhotic Incremental effectiveness: 0.0631 QALYs (Treatment 15.3308 QALYs, Comparator 15.2677 QALYs)  Non-first treatment patients with genotype 1a and cirrhotic Incremental effectiveness: 0.0507 QALYs (Treatment 13.9260 QALYs, Comparator 13.8753 QALYs)  First-treated patients with genotype 1b and noncirrhotic Incremental effectiveness: 0.0250 QALYs (Treatment 15.3343 QALYs, Comparator 15.3094 QALYs)  First-treated patients with genotype 1b and cirrhotic Incremental effectiveness: 0.1538 QALYs (Treatment 14.0203 QALYs, Comparator 13.8665 QALYs)  Non-first treatment patients with genotype 1b and noncirrhotic Incremental effectiveness: 0.1094 QALYs (Treatment 15.3771 QALYs, Comparator 15.2677 QALYs)  Non-first treatment patients with genotype 1b and cirrhotic Incremental effectiveness: 0.1756 QALYs (Treatment 14.0203 QALYs, Comparator 13.8447 QALYs) | First-treated patients with genotype 1a and noncirrhotic Incremental costs: US$ -20078 (Treatment US$ 62337, Comparator US$ 82416)  First-treated patients with genotype 1a and cirrhotic Incremental costs: US$ -37815 (Treatment US$ 78201, Comparator US$116016)  Non-first treatment patients with genotype 1a and noncirrhotic Incremental costs: US$ -38086 (Treatment US$ 62337, Comparator US$ 100423)  Non-first treatment patients with genotype 1a and cirrhotic Incremental costs: US$ -101906 (Treatment US$ 78201, Comparator US$ 180107)  First-treated patients with genotype 1b and noncirrhotic Incremental costs: US $ -22658 (Treatment US$ 59758, Comparator US$ 82416)  First-treated patients with genotype 1b and cirrhotic Incremental costs: US$ -41459 (Treatment US$ 74557, Comparator US$ 116016)  Non-first treatment patients with genotype 1b and noncirrhotic Incremental costs: US$ -41312 (Treatment US$ 59111, Comparator US$ 100423)  Non-first treatment patients with genotype 1b and cirrhotic Incremental costs: US$ -105908 (Treatment US$74557, Comparator US$ 180465) | First-treated patients with genotype 1a and noncirrhotic Incremental costs: US$ -21738.82 (Treatment US$ 67490.07, Comparator US$ 89228.89)  First-treated patients with genotype 1a and cirrhotic Incremental costs: US$ -40940.97 (Treatment US$ 84665.46, Comparator US$125606.43)  Non-first treatment patients with genotype 1a and noncirrhotic Incremental costs: US$ -41234.36 (Treatment US$ 67490.07, Comparator US$ 108724.43)  Non-first treatment patients with genotype 1a and cirrhotic Incremental costs: US$ -110330.03 (Treatment US$ 84665.46, Comparator US$ 194995.49)  First-treated patients with genotype 1b and noncirrhotic Incremental costs: US $ -24531.01 (Treatment US$ 64697.88, Comparator US$ 89228.89)  First-treated patients with genotype 1b and cirrhotic Incremental costs: US$ -44886.20 (Treatment US$ 80720.23, Comparator US$ 125606.43)  Non-first treatment patients with genotype 1b and noncirrhotic Incremental costs: US$ -44727.04 (Treatment US$ 63997.39, Comparator US$ 108724.43)  Non-first treatment patients with genotype 1b and cirrhotic Incremental costs: US$ -114662.85 (Treatment US$80720.23, Comparator US$ 195383.08) | Regardless of whether it is combined with liver cirrhosis or has undergone standard treatment in the past, for all patients with gene type 1a and 1b, the treatment group have lower cost and higher effectiveness than the control group. ICERs are negative and have an absolute advantage. |  |  |
|  | EBR/GZR vs. SOF/VEL | First-treated patients with genotype 1a and noncirrhotic Incremental effectiveness: 0.0137 QALYs (Treatment 15.3308 QALYs, Comparator 15.3171 QALYs)  First-treated patients with genotype 1a and cirrhotic Incremental effectiveness: -0.0938 QALYs (Treatment 13.9260 QALYs, Comparator 14.0198 QALYs)  Non-first treatment patients with genotype 1a and noncirrhotic Incremental effectiveness: 0.0137 QALYs (Treatment 15.3308 QALYs, Comparator 15.3171 QALYs)  Non-first treatment patients with genotype 1a and cirrhotic Incremental effectiveness: -0.0938 QALYs (Treatment 13.9260 QALYs, Comparator 14.0198 QALYs)  First-treated patients with genotype 1b and noncirrhotic Incremental effectiveness: -0.0422 QALYs (Treatment 15.3343 QALYs, Comparator 15.3765 QALYs)  First-treated patients with genotype 1b and cirrhotic Incremental effectiveness: 0.2157 QALYs (Treatment 14.0203 QALYs, Comparator 13.8046 QALYs)  Non-first treatment patients with genotype 1b and noncirrhotic Incremental effectiveness: 0.0006 QALYs (Treatment 15.3771 QALYs, Comparator 15.3765 QALYs)  Non-first treatment patients with genotype 1b and cirrhotic Incremental effectiveness: 0.2157 QALYs (Treatment 14.0203 QALYs, Comparator 13.8046 QALYs) | First-treated patients with genotype 1a and noncirrhotic Incremental costs: US$ -18046 (Treatment US$ 62337, Comparator US$ 80383)  First-treated patients with genotype 1a and cirrhotic Incremental costs: US$ -16729 (Treatment US$ 78201, Comparator US$ 94930)  Non-first treatment patients with genotype 1a and noncirrhotic Incremental costs: US$ -18046 (Treatment US$62337, Comparator US$ 80383)  Non-first treatment patients with genotype 1a and cirrhotic Incremental costs: US$ -16729 (Treatment US$ 78201, Comparator US$ 94930)  First-treated patients with genotype 1b and noncirrhotic Incremental costs: US$ -19727 (Treatment US$ 59758, Comparator US$ 79485)  First-treated patients with genotype 1b and cirrhotic Incremental costs: US$ -22892 (Treatment US$ 74557, Comparator US$ 97449)  Non-first treatment patients with genotype 1b and noncirrhotic Incremental costs: US$ -20374 (Treatment US$ 59111, Comparator US$ 79485)  Non-first treatment patients with genotype 1b and cirrhotic Incremental costs: US$ -22892 (Treatment US$ 74557, Comparator US$ 97449) | First-treated patients with genotype 1a and noncirrhotic Incremental costs: US$ -19537.76 (Treatment US$ 67490.07, Comparator US$ 87027.83)  First-treated patients with genotype 1a and cirrhotic Incremental costs: US$ -18111.9 (Treatment US$ 84665.46, Comparator US$ 102777.36)  Non-first treatment patients with genotype 1a and noncirrhotic Incremental costs: US$ -19537.76 (Treatment US$67490.07, Comparator US$ 87027.83)  Non-first treatment patients with genotype 1a and cirrhotic Incremental costs: US$ -18111.9 (Treatment US$ 84665.46, Comparator US$ 102777.36)  First-treated patients with genotype 1b and noncirrhotic Incremental costs: US$ -21357.72 (Treatment US$ 64697.88, Comparator US$ 86055.60)  First-treated patients with genotype 1b and cirrhotic Incremental costs: US$ -24784.36 (Treatment US$ 80720.23, Comparator US$ 105504.59)  Non-first treatment patients with genotype 1b and noncirrhotic Incremental costs: US$ -22058.21 (Treatment US$ 63997.39, Comparator US$ 86055.60)  Non-first treatment patients with genotype 1b and cirrhotic Incremental costs: US$ -24784.36 (Treatment US$ 80720.23, Comparator US$ 105504.59) | For patients with genotype 1a and cirrhotic, whether or not they have undergone standard treatment in the past, the ICERs are US$ 193090.62/QALY.  For patients with genotype 1a and noncirrhotic, whether or not they have undergone standard treatment in the past, the treatment group have lower cost and higher utility than the control group. ICERs are negative, which have an absolute advantage.  For first-treated patients with genotype 1b and noncirrhotic, the ICER is US$ 506107.11/QALY.  For genotype 1b, patients with first-treated cirrhosis, non-first treatment noncirrhosis, and non-first treatment cirrhosis, the treatment group have lower cost and higher effectiveness than the control group. ICERs are negative, which have an absolute advantage. |  |  |
| Elbasha et al., 2017a | EBR/GZR vs. no treatment | Incremental effectiveness: 2.6517 QALYs (Treatment 11.5716 QALYs, Comparator 8.9199 QALYs) | Incremental costs: US$35006 (Treatment US$ 191242, Comparator US$ 156236) | Incremental costs: US$38313.88 (Treatment US$ 209313.35, Comparator US$ 170999.47) | US$ 14448.80/QALY | US$ 109449.47/QALY | Results robust |
|  | EBR/GZR vs. PegIFN/RBV | Incremental effectiveness: 1.2859 QALYs (Treatment 11.5716 QALYs, Comparator 10.2857 QALYs) | Incremental costs: US$ 4541 (Treatment US$ 191242, Comparator US$ 186701) | Incremental costs: US$ 4970.10 (Treatment US$ 209313.35, Comparator US$ 204343.25) | US$ 3865.08/QALY |  |  |
| Elbasha et al., 2017b | EBR/GZR with RAVs vs. EBR/GZR without RAVs | First-treated patients with noncirrhotic Incremental effectiveness: 0.0862 QALYs (Treatment 14.2458 QALYs, Comparator 14.1596 QALYs)  First-treated patients with cirrhotic Incremental effectiveness: 0.1803 QALYs (Treatment 12.9707 QALYs, Comparator 12.7904 QALYs)  Non-first treatment patients with noncirrhotic Incremental effectiveness: 0.0862 QALYs (Treatment 14.2458 QALYs, Comparator 14.1596 QALYs)  Non-first treatment patients with cirrhotic Incremental effectiveness: 0.1803 QALYs (Treatment 12.9707 QALYs, Comparator 12.7904 QALYs) | First-treated patients with noncirrhotic Incremental costs: US$ 2196  (Treatment US$ 62013, Comparator US$ 59817)  First-treated patients with cirrhotic Incremental costs: US$ 1289  (Treatment US$ 74659, Comparator US$ 73370)  Non-first treatment patients with noncirrhotic Incremental costs: US$ 2196 (Treatment US$ 62013, Comparator US$59817)  Non-first treatment patients with cirrhotic Incremental costs: US$ 1290  (Treatment US$ 74660, Comparator US$ 73370) | First-treated patients with noncirrhotic Incremental costs: US$ 2403.51  (Treatment US$ 67872.90, Comparator US$ 65469.39)  First-treated patients with cirrhotic Incremental costs: US$ 1410.81  (Treatment US$ 81713.88, Comparator US$ 80303.07)  Non-first treatment patients with noncirrhotic Incremental costs: US$ 2403.51 (Treatment US$ 67872.90, Comparator US$ 65469.39)  Non-first treatment patients with cirrhotic Incremental costs: US$ 1411.90  (Treatment US$ 81714.97, Comparator US$ 80303.07) | For patients with first-treated noncirrhosis, first-treated cirrhosis, non-first treatment noncirrhosis and non-first treatment cirrhosis, the ICERs are US$ 27882.95/QALY, US$ 7824.79/QALY, US$27882.95/QALY and US$ 7830.84/QALY, respectively. | US$54724.73/QALY | The drug prices and SVR rates of the two treatment options have an impact on the results. |
|  | EBR/GZR with RAVs vs. 3D regimen | First-treated patients with noncirrhotic Incremental effectiveness: 0.0667 QALYs (Treatment 14.2458 QALYs, Comparator 14.1791 QALYs)  First-treated patients with cirrhotic Incremental effectiveness: 0.2801 QALYs (Treatment 12.9707 QALYs, Comparator 12.6906 QALYs)  Non-first treatment patients with noncirrhotic Incremental effectiveness: 0.0528 QALYs (Treatment 14.2458 QALYs, Comparator 14.1930 QALYs)  Non-first treatment patients with cirrhotic Incremental effectiveness: 0.2269 QALYs (Treatment 12.9707 QALYs, Comparator 12.7438 QALYs) | First-treated patients with noncirrhotic Incremental costs: US$ -13658  (Treatment US$ 62013, Comparator US$ 75651)  First-treated patients with cirrhotic Incremental costs: US$ -84586  (Treatment US$ 74659, Comparator US$ 159245)  Non-first treatment patients with noncirrhotic Incremental costs: US$ -13825 (Treatment US$62013, Comparator US$ 75838)  Non-first treatment patients with cirrhotic Incremental costs: US$ -84135 (Treatment US$ 74660, Comparator US$ 158795) | First-treated patients with noncirrhotic Incremental costs: US$ -14948.61  (Treatment US$ 67872.90, Comparator US$ 82799.61)  First-treated patients with cirrhotic Incremental costs: US$ -92578.92  (Treatment US$ 81713.88, Comparator US$ 174292.80)  Non-first treatment patients with noncirrhotic Incremental costs: US$ -15131.39 (Treatment US$67872.90, Comparator US$ 83004.29)  Non-first treatment patients with cirrhotic Incremental costs: US$ -92085.31 (Treatment US$ 81714.97, Comparator US$ 173800.28) | Regardless of whether it is combined with liver cirrhosis, or has undergone standard treatment in the past, the treatment group have lower cost and higher effectiveness than the control group, and ICERs are negative, which have an absolute advantage. |  |  |
|  | EBR/GZR with RAVs vs. LDV/SOF with 12weeks | First-treated patients with noncirrhotic Incremental effectiveness: -0.0081 QALYs (Treatment 14.2458 QALYs, Comparator 14.2539 QALYs)  First-treated patients with cirrhotic Incremental effectiveness: 0.0151 QALYs (Treatment 12.9707 QALYs, Comparator 12.9556 QALYs)  Non-first treatment patients with noncirrhotic Incremental effectiveness: 0.0249 QALYs (Treatment 14.2458 QALYs, Comparator 14.2209 QALYs)  Non-first treatment patients with cirrhotic Incremental effectiveness: 0.1105 QALYs (Treatment 12.9707 QALYs, Comparator+RBV 12.8602 QALYs) | First-treated patients with noncirrhotic Incremental costs: US$ -34709 (Treatment US$ 62013, Comparator US$ 96722)  First-treated patients with cirrhotic Incremental costs: US$ -34992  (Treatment US$ 74659, Comparator US$ 109651)  Non-first treatment patients with noncirrhotic Incremental costs: US$ -35185 (Treatment US$ 62013, Comparator US$ 97198)  Non-first treatment patients with cirrhotic Incremental costs: US$ -40847  (Treatment US$ 74660, Comparator US$ 115507) | First-treated patients with noncirrhotic Incremental costs: US$ -37988.81 (Treatment US$ 67872.90, Comparator US$ 105861.71)  First-treated patients with cirrhotic Incremental costs: US$ -38298.55  (Treatment US$ 81713.88, Comparator US$ 120012.43)  Non-first treatment patients with noncirrhotic Incremental costs: US$ -38509.79 (Treatment US$ 67872.90, Comparator US$ 106382.69)  Non-first treatment patients with cirrhotic Incremental costs: US$ -44706.82  (Treatment US$ 81714.97, Comparator US$ 126421.79) | For patients with first-treated noncirrhosis, the ICER is US$4689976.54/QALY.  For patients with first-treated cirrhosis, non-first treatment noncirrhosis, and non-first treatment cirrhosis, the treatment group have lower cost and higher effectiveness than the control group. ICERs are negative, which have an absolute advantage. |  |  |

Note: ICER, incremental cost effectiveness ratios; QALY, quality-adjusted life year; EBR/GZR, elbasvir/grazoprevir; 3D, ombitasvir/paritaprevir/ritonavir+dasabuvir; SVR, sustained virological response; GLE/PIB, glecaprevir/pibrentasvir; SOF/VEL, sofosbuvir/velpatasvir; PegIFN/RBV, Pegylated interferon/ribavirin; DCV/ASV, daclatasvir/asunaprevir; LDV/SOF, ledipasvir/sofosbuvir; SOF, sofosbuvir; RBV, ribavirin; RAVs, resistance-associated variants.
